# JNK Signaling Pathway Suppresses LPS-Mediated Apoptosis of HK-2 Cells by Upregulating NGAL

**DOI:** 10.1155/2020/3980507

**Published:** 2020-04-24

**Authors:** Mei Han, Yuxia Pan, Mengying Gao, Junli Zhang, Fan Wang

**Affiliations:** ^1^Department of Emergency, The Second Hospital of Hebei Medical University, Shijiazhuang, China; ^2^Department of Hemopathy, The Second Hospital of Hebei Medical University, Shijiazhuang, China

## Abstract

**Objective:**

To explore the role of the c-Jun N-terminal kinase (JNK) signaling pathway in upregulated NGAL expression and its antiapoptotic mechanism in lipopolysaccharide (LPS)-mediated renal tubular epithelial cell injury.

**Methods:**

In vitro, HK-2 cells were divided into five groups (Con, LPS 1 h, LPS 3 h, LPS 6 h, and LPS 12 h groups) based on the time of LPS (10 *μ*M) treatment. NGAL and caspase-3 gene expression levels were detected by RT-PCR to assess dynamic changes. HK-2 cells were pretreated with SP600125 (20 *μ*M) for 2 hours, followed by LPS (10 *μ*M) stimulation for 3 hours. NGAL and caspase-3 gene expression levels were then determined.

**Results:**

NGAL mRNA was increased significantly within 6 hours, and caspase-3 mRNA was increased within 3 hours after treatment (*P* < 0.05). Correlation analysis showed a high correlation between their expression (*r* = 0.448, *P* < 0.05). After pretreatment with SP600125, mRNA expression of NGAL in the LPS group was inhibited, while that of caspase-3 was increased significantly. The NGAL mRNA expression level in the SB + LPS group was decreased significantly compared with that in the LPS group, but it was slightly higher than that in the SP group (∼1.5 times of that in the Con group). However, caspase-3 mRNA expression was increased significantly in the SB + LPS group (*P* < 0.001) (3.5 times of that in the Con group). It also showed a significant increase compared with SP and LPS groups (*P* < 0.001 vs. SB group; *P* < 0.05 vs. LPS group). We also found that NGAL and caspase 3 proteins were increased significantly in LPS and SP + LPS groups, but SP600125 decreased the NGAL level by almost 35% and increased the caspase 3 level by 50% in the SP + LPS group compared with the LPS group (*P* < 0.05).

**Conclusions:**

The JNK signaling pathway inhibits LPS-mediated apoptosis of renal tubular epithelial cells by upregulating NGAL.

## 1. Introduction

Neutrophil gelatinase-associated lipocalin (NGAL) is a multifunctional protein expressed at very low levels under normal physiological conditions. However, when the body is damaged, its expression in epithelial cells of the kidney, colon, liver, and lungs increases dramatically [1]. Our previous study found that NGAL mRNA expression was upregulated significantly when HK-2 cells were stimulated by lipopolysaccharide (LPS), which remarkably inhibited upregulation of caspase-3 in cells and thus reduced apoptosis of damaged cells [2]. As an acute-phase protein, NGAL may inhibit injury and protect epithelial cells. However, the mechanism by which expression of NGAL is upregulated in renal tubular cells during sepsis remains unclear. In our current study, LPS was used to stimulate HK-2 cells, a proximal tubular cell line derived from the normal kidney, and to observe changes in mRNA expression of NGAL and caspase-3. In addition, JNK-specific inhibitor SP600125 was used to pretreat HK-2 cells to observe the effect of upregulated NGAL on crucial enzymes of apoptosis and to identify the signaling pathways involved in upregulating NGAL during LPS-mediated renal epithelial cell injury and their possible roles.

## 2. Materials and Methods

### 2.1. Materials

The immortalized human proximal tubule epithelial cell line HK-2 was purchased from Bioleaf (Shanghai, China). Other reagents included *Escherichia coli* lipopolysaccharide (E. coli O111B4; Sigma, MO, USA), premium fetal bovine serum (PAA, Austria), DMEM (Gibco, USA), JNK pathway inhibitor SP600125 (Selleck, USA), PrimeScript™ RT regent kit (Takara, Japan), and Power SYBR Green PCR Master Mix (Takara, Japan). NGAL protein in culture supernatants was measured by an enzyme-linked immunosorbent assay (ELISA) kit (R&D Systems; Minneapolis, MN, USA). Primary antibodies were rabbit monoclonal antibodies against caspase 3 (Santa Cruz Biotechnology, USA) and *β*-actin (Sigma).

### 2.2. Primer Sequences

Primers were searched for in PubMed GenBank as shown in Table 1. Primers were synthesized by Invitrogen (USA).

### 2.3. Experimental Methods

#### 2.3.1. Cell Culture

HK-2 cells were seeded at 5 × 10^7^ per T25 flask containing D-MEM/F12 medium with 10% fetal bovine serum, 100 U/ml penicillin, and 100 U/ml streptomycin and cultured at 37°C in a humidified atmosphere with 5% CO_2_. Cryopreservation, resuscitation, and passaging of cells were performed routinely.

#### 2.3.2. Measurement of NGAL and Caspase-3 mRNA Expression after LPS Treatment

HK-2 cells were seeded in a six-well plate at 5 × 10^5^ cells per well. After 12 hours of culture, they were divided into five groups, a control group (Con) and four LPS treatment groups (LPS 1 h, LPS 3 h, LPS 6 h, and LPS 12 h) in which the cells were treated with 10 *μ*M LPS for 1, 3, 6, and 12 hours, respectively. Measurements were repeated three times for each group. At each time point, the medium in the culture plate was discarded, and the plate was washed with PBS once. Then, 1 ml of precooled Trizol was added, followed by agitation. The cell suspension in each well was collected in EP tubes and stored at −80°C to detect mRNA expression of NGAL and caspase 3.

#### 2.3.3. Measurement of NGAL and Caspase-3 mRNA Expression and Protein Secretion after SP600125 Pretreatment

*(1) Culture and Treatment of HK-2 Cells*. HK-2 cells were seeded in a 6-well plate at 2 × 10^5^ cells per well and then cultured in DMEM/F12 medium containing 10% fetal bovine serum without antibiotics for 24 hours.

The cells were divided into four groups: control, LPS, SP, and SP + LPS groups. Each group had three replicates in three wells. The measurements were repeated three times for each group. SP600125 (20 *μ*mol/L) was added to the medium in SP and SP + LPS groups, and the cells were cultured for 2 hours. Subsequently, the culture medium was replaced with serum-free DMEM/F12 medium for Con and SP groups or D-MEM/F12 medium containing 10 *μ*M LPS for LPS and SP + LPS groups, and the cultures were continued for another 3 hours.

After the time points were reached, the medium in the culture plate was discarded and the plate was washed with PBS once. Then, 1 ml of Trizol was added, followed by agitation. The cell suspension in each well was collected in EP tubes and stored at −80°C to detect mRNA expression of NGAL and caspase 3.

*(2) Quantitative Real-Time Polymerase Chain Reaction (qRT-PCR)*. HK-2 cells collected from the plates were dissolved on ice in Trizol. After the cellular RNA was extracted, it was analyzed for purity using a Nanodrop ND-1000 spectrophotometer (Nanodrop Technologies, Wilmington, DE). cDNA was obtained using a reverse transcription kit, and 2 *μ*l cDNA was used for PCR amplification. PCR was performed under the following conditions: predenaturation at 95°C for 30 s, followed by denaturation at 95°C for 5 s, and annealing at 60°C for 34 s. The amplification was performed for 40 cycles. *β*-Actin was used as an internal reference gene. Relative gene expression was analyzed by the ΔΔCt method with the housekeeping gene GAPDH as the internal control.

*(3) ELISA*. HK-2 cells were treated with LPS and SP600125 as described above, and then the culture supernatant was collected. The concentration of NGAL in the culture supernatant was measured using the ELISA kit, according to the manufacturer's instructions.

*(4) Western Blotting*. Total proteins were extracted from cell lysates according to standard methods. Equal amounts of proteins were subjected to 10% SDS-PAGE and then transferred to a nitrocellulose membrane that was incubated with primary antibodies against caspase 3 and *β*-actin overnight, followed by a secondary goat anti-rabbit antibody. Finally, western blots were scanned, and semiquantitative analysis was performed using *β*-actin as the control for protein loading. Each experiment was repeated at least three times.

### 2.4. Statistical Analysis

Statistical analysis was performed using SPSS 17.0 software. Data are expressed as the mean ± standard deviation ($\bar{x}\pm \text{SD}$). After testing for normality in each group, one-way analysis of variance was used to compare the LSD means. *P* < 0.05 was regarded as statistically significant.

## 3. Results

### 3.1. Endotoxin Stimulation of HK-2 Cells Affects NGAL Expression and Apoptosis

**Figure 1:**
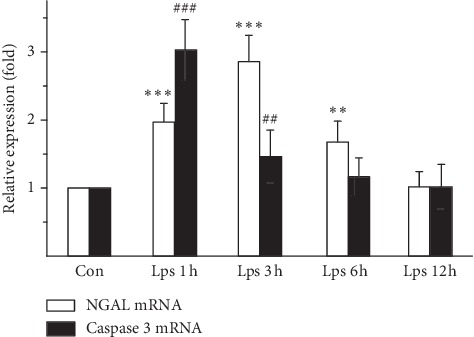
Expression of NGAL and caspase 3 mRNAs in LPS-treated HK-2 cells. Data are the mean ± S.D. of three separate experiments performed in duplicate. There was a 2.0-fold increase in NGAL mRNA expression at 1 hour after 10 *μ*M LPS treatment, which increased to 2.8-fold at 3 hours after 10 *μ*M LPS treatment and then decreased to 1.6-fold at 3 hours after LPS treatment. ^*∗∗*^*P* < 0.01 and ^*∗∗∗*^*P* < 0.001, relative to the control group. There was a 3.02-fold increase in caspase-3 mRNA expression at 1 hour after 10 *μ*M LPS treatment, decreasing to 1.4-fold at 3 hours after 10 *μ*M LPS treatment. ^##^*P* < 0.01 and ^###^*P* < 0.001, relative to the control group.

As measured by qRT-PCR, after HK-2 cells were treated with 10 *μ*M LPS, NGAL mRNA expression was increased significantly within 6 hours after stimulation and significantly different in LPS 1 h, LPS 3 h, and LPS 6 h groups compared with the Con group (LPS 1 h and 3 h groups vs. Con group, *P* < 0.001; LPS 6 h group vs Con group, *P* < 0.01). At 12 hours after LPS treatment, mRNA expression of NGAL returned to the baseline level, showing no significant difference compared with the Con group (*P* > 0.05). The peak level of NGAL mRNA expression in HK-2 cells was 2.856 ± 0.389 times higher than the baseline in the LPS 3 h group. After HK-2 cells were treated with 10 *μ*M LPS, caspase mRNA expression was significantly higher within 3 hours after stimulation and significantly different in LPS 1 h and LPS 3 h groups compared with the Con group (LPS 1 h group vs Con group, *P* < 0.001; LPS 3 h group vs Con group, *P* < 0.01). At 6 hours after LPS treatment, mRNA expression of caspase 3 returned to the baseline level, and caspase-3 mRNA expression in LPS 6 h and LPS 12 h groups showed no significant difference compared with the Con group (*P* > 0.05). The peak level of caspase-3 mRNA expression in HK-2 cells was 3.029 ± 0.448 times higher than the baseline in the LPS 1 h group. Correlation analysis showed a high correlation between NGAL and caspase-3 mRNA expression (*r* = 0.448, *P* < 0.05) (Figure 1).

### 3.2. Endotoxin Stimulation of HK-2 Cells Affects NGAL Expression and Apoptosis after Pretreatment with SP600125

**Figure 2:**
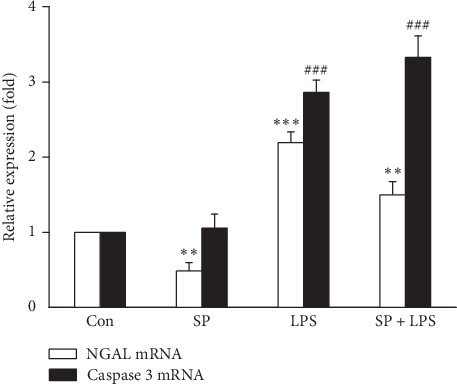
Expression of NGAL and caspase-3 mRNAs in LPS-treated HK-2 cells after SP600125 pretreatment. Data are the mean ± S.D. of three separate experiments performed in duplicate. ^*∗∗*^*P* < 0.01 and ^*∗∗∗*^*P* < 0.001, relative to the NGAL mRNA control group. ^##^*P* < 0.01 and ^###^*P* < 0.001, relative to the caspase-3 mRNA control group.

**Figure 3:**
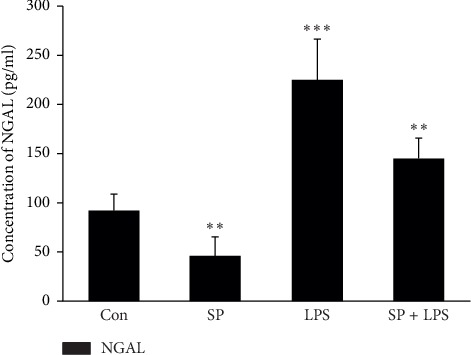
JNK inhibitor SP600125 (20 *μ*M) attenuates LPS-induced NGAL protein secretion in HK-2 cells. Data are the mean ± S.D. of three separate experiments performed in duplicate. ^*∗∗*^*P* < 0.01 and ^*∗∗∗*^*P* < 0.001, relative to the control group.

**Figure 4:**
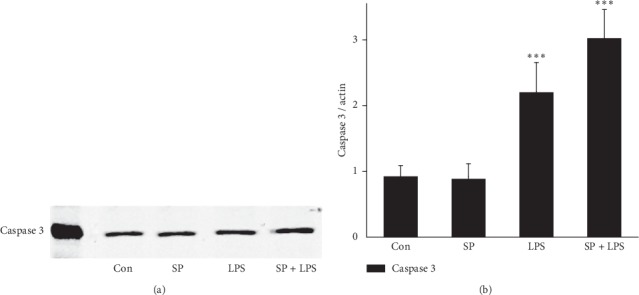
JNK inhibitor SP600125 (20 *μ*M) increases LPS-induced caspase 3 protein expression in HK-2 cells. (a) Western blot of caspase 3 protein. (b) Quantification of caspase 3 protein levels. *β*-Actin was used as an internal control. Data are the mean ± S.D. of three separate experiments performed in duplicate. ^*∗∗*^*P* < 0.01 and ^*∗∗∗*^*P* < 0.001, relative to the control group.

After pretreatment with SP600125, mRNA expression of NGAL in LPS-stimulated HK-2 cells was inhibited, while mRNA expression of caspase-3 was increased significantly. NGAL mRNA expression in the LPS group was increased significantly (*P* < 0.001) by 2.0 times of that in the Con group. Moreover, caspase-3 mRNA expression was upregulated significantly (*P* < 0.01) by 2.8 times of that in the Con group. NGAL mRNA expression in the SP group was inhibited significantly (*P* < 0.01) to 41% of that in the Con group. However, caspase-3 mRNA expression showed no significant change compared with the Con group (*P* > 0.05. The NGAL mRNA expression level in the SB + LPS group was decreased significantly compared with that in the LPS group, but it was slightly higher than that in the SP group (∼1.5 times of that in the Con group). However, caspase-3 mRNA expression was increased significantly in the SB + LPS group (*P* < 0.001) by 3.5 times of that in the Con group. It also showed a significant increase compared with SP and LPS groups (*P* < 0.001 vs. SB group; *P* < 0.05 vs. LPS group) (Figure 2). We also determined concentrations of NGAL and caspase 3 proteins in HK-2 cells and culture supernatants. Increased levels of NGAL and caspase 3 were observed in LPS and SP + LPS groups, but SP600125 decreased the NGAL level in the SP + LPS group by almost 35% compared with the LPS group (*P* < 0.05, Figure 3) and increased the caspase 3 level by around 50% (*P* < 0.05, Figure 4). These observations suggest that JNK is involved in LPS-induced NGAL expression and apoptosis of HK-2 cells.

## 4. Discussion

NGAL was discovered by Kjeklsen et al. [3, 4] in 1993 during a study on matrix metalloproteinase 9 (also known as 92 kDa gelatinase). Subsequently, a series of studies have demonstrated that NGAL is a good biomarker for early diagnosis of acute kidney injury (AKI) [5]. However, the biological role of NGAL as an acute-phase protein in epithelial cells during renal injury remains unclear. Mishra et al. applied exogenous NGAL to ischemic AKI rat models and found that it protected renal proximal tubular epithelial cells, alleviated ischemia-reperfusion injury, and suppressed apoptosis after injury [6]. However, few studies have explored the role of NGAL in protecting the kidneys during septic AKI and its relevant mechanism. In our previous in vitro study [2], apoptosis was the main pathological feature of LPS-mediated HK-2 cell injury. Timely upregulated expression of the NGAL gene in damaged renal tubular epithelial cells inhibited upregulation of the caspase-3 gene, thereby suppressing apoptosis and alleviating cellular damage. However, the signaling pathway regulating NGAL was unclear.

Endotoxin is the main component of the Gram-negative bacterial cell wall, which activates toll-like receptor 4 in renal tubular epithelial cells. It also induces the expression of various inflammatory cytokines and chemokines through the NF-*κ*B pathway, leading to AKI, and mediates inflammatory injury by activating the mitogen-activated protein kinase (MAPK) family [7]. As a serine-threonine protein kinase signal transduction system, MAPK is an important transmembrane signaling pathway in cells. It has four family members: extracellular signal-regulated kinase (ERK), stress-activated protein kinase JNK (JNK), p38 MAPK, and ERK5 [8]. Recent studies have found that activation of JNK upregulates the synthesis of core inflammatory factors, such as TNF-*α*, IL-6, and IL-1*β*, in endotoxic shock rat models, and promotes inflammatory factors and further activates JNK, thereby amplifying endotoxin-induced damage. Inhibition of the JNK/MAPK signaling pathway significantly reduces the mortality rate of sepsis [9, 10]. SP600125 used in our current study is a specific inhibitor of the JNK/MAPK signaling pathway. It inhibits the kinase by competing with JNK for the ATP-binding site. In our study, we inhibited the JNK pathway with SP100125 (20 *μ*M), followed by LPS stimulation. Next, we observed changes in NGAL and caspase-3 expression of HK-2 cells to investigate the role of the JNK pathway in the inhibitory effect of NGAL on renal tubular epithelial cell injury. The results confirmed that SP600125 inhibited the upregulation of NGAL in HK-2 cells after LPS injury by blocking the JNK pathway. This result was similar to the study by Konno et al. who showed that NGAL mRNA expression was downregulated by SP600125 (10 *μ*M) in IL-1*β*-induced canine renal tubular cells [11]. In addition, the expression of caspase-3, one of the crucial enzymes in activating apoptosis, was increased, indicating that apoptosis would be promoted in LPS-treated renal tubular epithelial cells. These findings suggest that regulating the expression of NGAL in the kidneys through the JNK signaling pathway inhibits apoptosis and attenuates endotoxin-induced renal damage, which may be a new approach for the treatment of acute kidney injury associated with sepsis.

## Figures and Tables

**Table 1:** Primers used for RT-PCR.

Gene	Sequence	Product (bp)
NGAL	TTGGGACAGGGAAGACGA	240
	TCACGCTGGGCAACATTA	

Caspase 3	GTTCATCCAGTCGCTTTGTGC	110
	AAATTCTGTTGCCACCTTTCG	

GAPDH	TCGCGGGAGACCACCGACAC	258
	GGGGTGTTGGGTCAGGTCTCTG	

## Data Availability

All data generated or analyzed during this study are included within the article.
